# Interleukin-1β secretion induced by mucosa-associated gut commensal bacteria promotes intestinal barrier repair

**DOI:** 10.1080/19490976.2021.2014772

**Published:** 2022-01-06

**Authors:** Wan-Jung H. Wu, Myunghoo Kim, Lin-Chun Chang, Adrien Assie, Fatima B. Saldana-Morales, Daniel F. Zegarra-Ruiz, Kendra Norwood, Buck S. Samuel, Gretchen E. Diehl

**Affiliations:** a Immunology Graduate Program, Baylor College of Medicine, Houston, TX, USA; b Memorial Sloan Kettering Cancer Center, Immunology Program of the Sloan Kettering Institute, New York, NY, USA; c Department of Molecular Virology and Microbiology, Baylor College of Medicine, Houston, TX, USA; d Present Address: Department of Animal Science, College of Natural Resources and Life Sciences, Pusan National University, Miryang, Korea; e Neuroscience Graduate Program, Baylor College of Medicine, Houston, TX, USA

**Keywords:** Microbiota, intestinal barrier repair, CX_3_CR1^+^ MNPS, ILC3s, IL-22, IL-1β, colitis

## Abstract

The gut microbiota is essential for maintenance and repair of the intestinal epithelial barrier. As shifts in both intestinal epithelial barrier function and microbiota composition are found in inflammatory bowel disease patients, it is critical to understand the role of distinct bacteria in regulating barrier repair. We identified a mouse commensal *E. coli* isolate, GDAR2-2, that protects mice from *Citrobacter rodentium* infection and dextran sulfate sodium-induced colitis. Colonization with GDAR2-2 in mice resulted in expansion of CX_3_CR1^+^ mononuclear phagocytes, including CX_3_CR1^+^ macrophages/dendritic cells and monocytes, along with IL-22-secreting type 3 innate lymphoid cells and improved epithelial barrier function. In vitro co-culture of macrophages with GDAR2-2 resulted in IL-1β production. In vivo, protection after GDAR2-2 colonization was lost after depletion of CX_3_CR1^+^ MNPs, or blockade of IL-1β or IL-22. We further identified human commensal *E. coli* isolates that similarly protect mice from *C. rodentium* infection through CX_3_CR1^+^ MNP and IL-1β production. Together, these findings demonstrate an unexpected role for commensal bacteria in promoting IL-1β secretion to support intestinal barrier repair.

## Introduction

Commensal bacteria have coevolved with their hosts and support multiple host functions including digestion and absorption of nutrients, modulation of immune responses, maintenance of the epithelium and regulation of intestinal motility.^1^ The greatest load of microbes resides within the large intestine, where a single layer of epithelial cells forms a barrier to separate intestinal contents from host internal tissues.^2,3^ Thus, protection and repair of this barrier are key to maintaining digestion as well as limiting entry of intestinal microbes into the gut tissue. Signals from the microbiota support epithelial functions with increased pathology in models of epithelial damage in germ-free or antibiotic-treated mice.^4–6^ Further, altered interactions between host and microbiota can exacerbate intestinal inflammation and underlie chronic inflammatory disorders including inflammatory bowel diseases (IBD).^2,7–9^

Barrier repair is supported by the immune system. Mononuclear phagocytes (MNPs) expressing the chemokine receptor CX_3_CR1 are central regulators of this process,^10,11^ with multiple functions regulated by the microbiota.^10–13^ CX_3_CR1^+^ MNPs arise from Ly6C^hi^ monocyte precursors that differentiate within the tissue into dendritic cells (DCs) or macrophages.^14–16^ In the steady state, lamina propria CX_3_CR1^+^ MNPs are highly phagocytic and clear apoptotic cells, cellular debris, and microbes.^17^ After microbial exposure, CX_3_CR1^+^ MNPs produce limited pro-inflammatory cytokines such as IL-6 or TNF but produce high amounts of the anti-inflammatory cytokine IL-10.^12,18,19^ During intestinal injury, microbial signals drive IL-1β and IL-23 production from CX_3_CR1^+^ MNPs which activates type 3 innate lymphoid cells (ILC3s) to secret IL-22. This pathway supports barrier repair and anti-microbial defenses.^10,20–23^ IL-22 induces epithelial cell proliferation, survival,^24,25^ and production of anti-microbial peptides (AMPs), such as RegIIIβ and RegIIIγ.^21^ IL-22 also regulates goblet cell regeneration and mucus secretion.^21,26^

Numerous studies describe changes in the intestinal microbiota composition, also known as dysbiosis, that are associated with inflammatory diseases. In IBD, decreased Bacteroidetes along with increased mucosa-associated bacteria including Proteobacteria,^7–9^ a phylum including *E. coli*, are found. This increases opportunity for direct interaction between the microbes and host tissue potentially impacts disease severity. Similar microbial shifts are also observed in diseases such as arthritis, diabetes, and asthma.^27–29^ As the microbiota orchestrates the development and functions of the immune system, changes in bacterial composition are thought to underlie inflammatory disorders. However, the role of specific microbes in such diseases are still being determined.

Here, we identified a mouse commensal *E. coli* isolate, GDAR2-2, that protects mice from increased damage after either *Citrobacter rodentium* infection or dextran sulfate sodium (DSS) treatment. After depleting the microbiota with antibiotics, colonization with GDAR2-2 promotes CX_3_CR1^+^ MNP expansion in the intestine, which then amplifies IL-22 secretion by type 3 innate lymphoid cells (ILC3s) and promotes transit-amplifying cell proliferation. Unexpectedly, this protection depends on IL-1β but not IL-23 production. Blockade of IL-22 also results in loss of protection. We further identified additional human intestinal *E. coli* that induce IL-1β production and offer similar intestinal protection. These findings highlight the pro-inflammatory pathways activated by intestinal microbes can be critical to limit intestinal damage and promote barrier repair.

## Results

### Commensal microbiota protects mice in models of colitis

**Figure 1.**
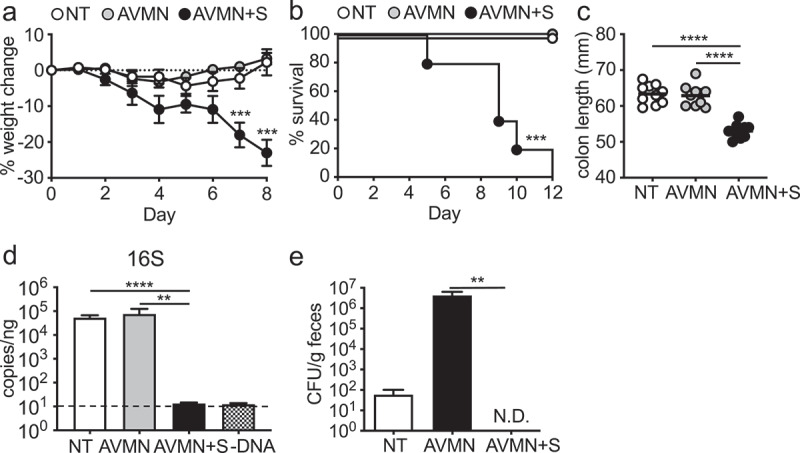
AVMN-resistant mouse commensal bacteria protects mice from *C. rodentium* infection. (a-c) WT C57BL/6 (B6) mice treated with AVMN, AVMN+S or left untreated were infected with *C. rodentium*. (a) weight change, (b) survival rate, and (c) colon length at day 12 post infection. (d-e) AVMN-resistant mouse commensal bacteria identified in feces. (d) Total fecal bacteria as detected by qPCR. (e) Bacteria colony forming units (CFU) as determined by plating on blood agar plates. Data are representative from at least 2 experimental repeats. (a,d,e) Data are shown as mean ± SEM compared by one-way ANOVA with Bonferroni correction or (b) log rank test. (c) Data points are individual mice with mean as compared by one-way ANOVA with Bonferroni correction. **p ≤ 0.01, *** p ≤ 0.001, **** p ≤ 0.0001. none detected (n.d.). Dotted line represents limit of detection for assay.

We and others previously showed that the microbiota promotes barrier repair and is protective in models of colitis.^10^ To better understand protective signals induced by the microbiota, we treated wild type (WT) mice with the broad-spectrum antibiotics ampicillin, vancomycin, metronidazole and neomycin (AVMN) cocktail to deplete the microbiota and infected mice with *C. rodentium*, a model of attaching and effacing (A/E) *E. coli* infection.^21,30^ Unlike previous reports,^4,31^ treatment with AVMN did not alter susceptibility to *C. rodentium* infection as demonstrated by no increase in weight loss, mortality, or decrease in colon length (Figure 1(a-c)). To test whether this regime depleted all intestinal microbes, we utilized qPCR to detect 16s rRNA and found no reduction in intestinal microbes (Figure 1(d)). We then treated mice with AVMN in combination with streptomycin (AVMN+S) prior to administration of *C. rodentium*. As assessed by qPCR, this treatment was sufficient to deplete intestinal microbes below the level of detection (Figure 1(d)). Upon infection with *C. rodentium*, AVMN+S-treated mice showed increased susceptibility to *C. rodentium* with increased weight loss and shorter colon length, indicating more severe intestinal inflammation (Figure 1(a-c)).

To identify the microbe(s) that offer protection after AVMN treatment, we cultured fecal microbes from AVMN-treated mice under aerobic and anaerobic conditions. As predicted by qPCR analysis, we found bacterial growth from AVMN but not AVMN+S-treated mice with equivalent growth under both aerobic and anaerobic conditions (Figure 1(e) and data not shown).

We isolated 20 colonies from the AVMN-treated group and identified all as *E. coli* by sequencing small subunit ribosomal 16S gene amplicons and serotyping as O112:H8. We further confirmed that all isolates were resistant to the AVMN antibiotic cocktail but remained susceptible to streptomycin and gentamicin. By PCR or culture of colonic contents from AVMN treated mice we were unable to identify outgrowth of any additional bacteria.

To further characterize the *E. coli*, we selected one isolate for genomic sequencing. The complete genome of the B1 phylotype, mouse derived *E. coli* isolate GDAR2-2 was sequenced using PacBio and assembled *de novo* into two fully closed contigs. This consisted of the bacterial genome of 4,928,781 base pairs (bp) in length with a 50.73% GC content. We also identified an associated 71,810 bp IncF low copy number plasmid with 94 predicted coding genes with mostly hypothetical functions. Sequence annotations of the genome predicted 4841 coding sequences (Figure S1a) including the presence of genes commonly found associated with commensal *E. coli*. We also found adhesion genes for long polar fimbriae and pilus assembly proteins.

To test whether treatment with individual antibiotics were sufficient to disrupt gut commensal bacteria colonization, we utilized ampicillin alone and ampicillin in combination with streptomycin. Treatment with ampicillin and streptomycin was sufficient to reduce intestinal bacteria load to a level below qPCR detection (Figure S2a). We utilized this antibiotic combination (ABX) in subsequent experiments.

**Figure 2.**
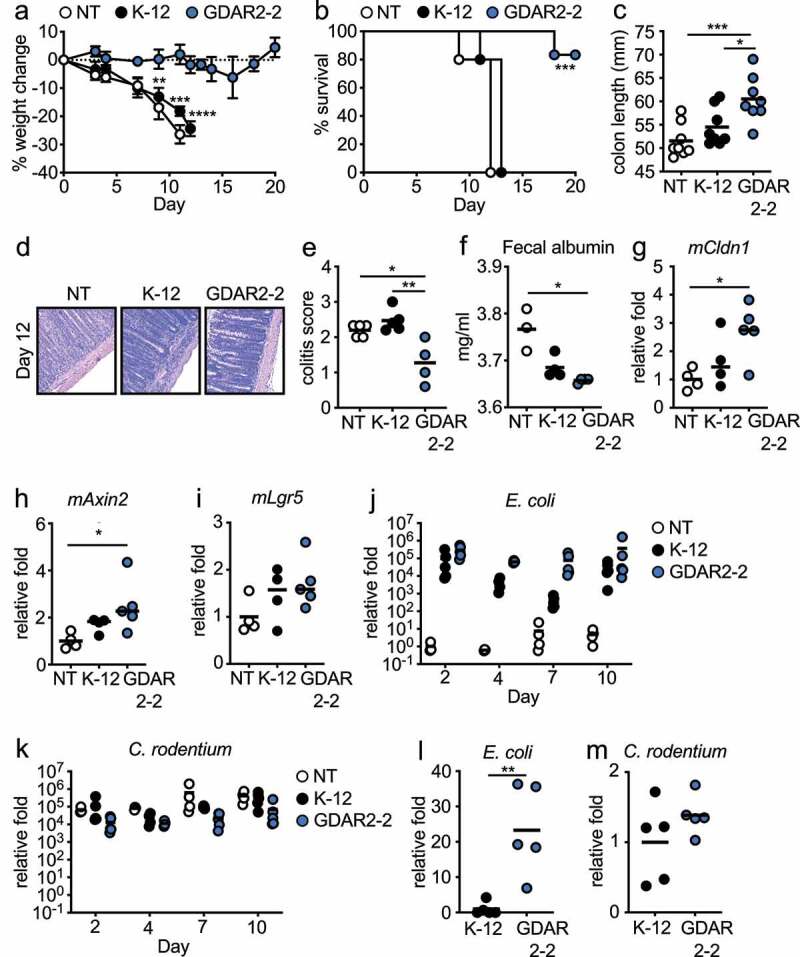
Mouse commensal *E. coli* isolate GDAR2-2 protects mice from *C. rodentium* infection. (a-i) Ampicillin and streptomycin (ABX)-treated B6 mice were colonized with lab strain *E. coli* K-12, GDAR2-2 or left uncolonized and further infected with *C. rodentium*. (a) weight change, (b) survival rate, (c) colon length 2 weeks after infection. (d-e) Representative H&E staining and colitis scores of colon. (f) albumin concentration in feces was measured at 4 days post infection. Expression of (g) mCldn1, (h) mAxin2 and (i) mLgr5 in colon 4 days after infection. Feces were collected at indicated time points for (j) *E. coli* and (k) *C. rodentium* quantification of bacterial load by qPCR. (l-m) 3 days after infection, colon tissues were collected and washed. Mucosa-associated (l) *E. coli* and (m) *C. rodentium* were determined by qPCR. Data are representative of at least 2 independent experiments. (a) Data are shown in mean ± SEM (a) or individual mice (c, e-m) and compared by one-way ANOVA with Bonferroni correction (a, c, e, h-k), Kruskal-Wallis with Dunn’s multiple comparison (f-g), log rank test (b), or Student’s t test (l-m). *p ≤ 0.05, **p ≤ 0.01, *** p ≤ 0.001, **** p ≤ 0.0001.

To confirm colonization with GDAR2-2 was sufficient to protect mice from *C. rodentium* infection, we treated mice with ABX and then left them uncolonized or colonized with the lab adapted *E. coli* strain MG1655 (K-12) or GDAR2-2 before infection with *C. rodentium*. Uncolonized mice or mice colonized with K-12 were equally susceptible to *C. rodentium* infection with equivalent weight loss, decreased survival, shorter colon lengths, and enhanced severity of intestinal pathology (Figure 2(a-e)). In contrast, GDAR2-2 colonized mice were protected from *C. rodentium* infection with reduced weight loss, increased survival, longer colon length, and ameliorated pathology with lower histological colitis scores after *C. rodentium* infection (Figure 2(a-e)). Supporting improved barrier function in GDAR2-2 colonized mice, unlike K-12, we found lower fecal albumin and increased expression of the tight junction protein, *Cldn1*, in the intestine of GDAR2-2 colonized mice as compared to uncolonized mice (Figure 2(f-g)).

Regeneration of intestinal epithelium occurs by inducing proliferation of transit-amplifying cells^32^ or by induction of Wnt agonist that promotes stem cell proliferation^33,34^ with both pathways shown to play a role in protection from *C. rodentium* infection.^33,34^ To determine if either of these pathways was activated after GDAR2-2 colonization, we utilized qPCR and found increased expression of transit-amplifying cell marker, Axin2 in the colon of GDAR2-2-colonized *C. rodentium* infected mice (Figure 2(h)). In contrast, we did not find activation of the Wnt pathway, as expression of the Wnt agonist, Rspo3, was similar between untreated, K-12, and GDAR2-2 colonized *C. rodentium* infected mice (Figure S3a). Further, the expression of Lgr5, a stem cell marker and Wnt target gene, was also not upregulated in GDAR2-2 colonized mice (Figure 2(i)). We also found increased expression of *Ascl2* in GDAR2-2-colonized mice which could indicate epithelial differentiation in GDAR2-2 mediated protection^35^ (Figure S3b). These data suggest that GDAR2-2 colonization is sufficient to protect mice from *C. rodentium* infection by increasing proliferation of transit-amplifying cells.

To test if GDAR2-2 inhibited *C. rodentium* intestinal colonization, we determined the levels of *E. coli* and *C. rodentium* in feces and intestinal mucosa of ABX-treated mice colonized with K-12 or GDAR2-2. By analyzing relative bacterial abundance in feces, we found both *E. coli* colonized equivalently at day of infection with *C. rodentium* (Figure 2(j)). However, colonization with either *E. coli* did not affect *C. rodentium* colonization levels as we found comparable levels of *C. rodentium* in feces in all groups and timepoints (Figure 2(k)). To test if there were differences in colonization at the intestinal mucosa, we performed qPCR of mucosa-associated bacteria in the intestine and found higher levels of GDAR2-2 compared to K-12 (Figure 2(l)). Our findings supported genomic analyses indicating that GDAR2-2 carries adherence genes such as long polar fimbria and pilus assembly proteins, both of which could mediate epithelial adhesion.^36–38^ However, we found no difference in mucosa *C. rodentium* levels between K-12 and GDAR2-2-colonized mice (Figure 2(m)). Together this suggests that protection after GDAR2-2 colonization is not due to competition with *C. rodentium* as has been shown with other *E. coli* isolates.^39^

To determine if protection after GDAR2-2 colonization was specific for *C. rodentium* infection, we treated mice with ABX before treatment with DSS which causes damage to the intestinal epithelium.^40^ As with *C. rodentium* infection, mice treated with ABX succumbed to DSS-induced colitis with significant weight loss (Figure S4(a-b)). Pre-colonization with GDAR2-2 protected mice from increased weight loss with increased survival after DSS treatment (Figure S4(c-d)).

### Mouse commensal E. coli induces ILC3 IL-22 secretion and epithelium regeneration

**Figure 3.**
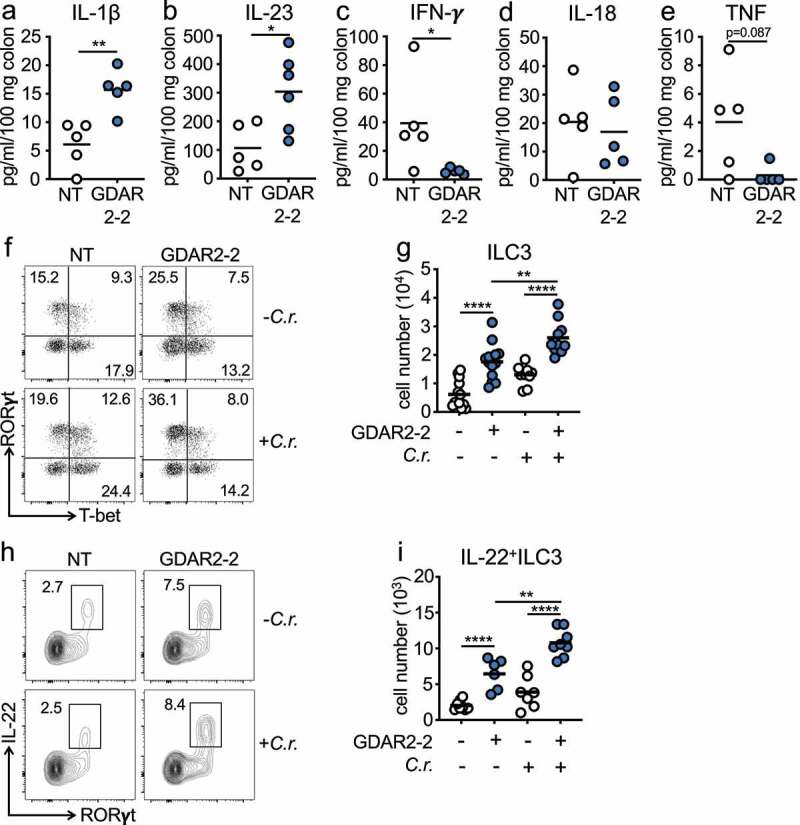
GDAR2-2 induces ILC3 and IL-22^+^ILC3 expansion. (a-e) ABX-treated B6 mice were colonized with GDAR2-2 or left un-colonized and infected with *C. rodentium*. 6 days after infection, colon secretion of (a) IL-1β, (b) IL-23, (c) IFN-γ, (d) IL-18 and (e) TNF was measured by LegendPlex. (f-i) ABX-treated B6 mice were colonized with GDAR2-2 or left un-colonized and infected with or without *C. rodentium*. Five days after infection, ILC3s and IL-22^+^ILC3s from large intestine laminar propria were isolated and analyzed by flow cytometry. (f) Representative dot plots of live lin^−^Eomes^−^CD90^+^ cells and (g) absolute number of live lin^−^Eomes^−^CD90^+^RORγt^+^ILC3. (h) Representative dot plots of live lin^−^Eomes^−^CD90^+^ cells and (i) absolute number of live lin^−^Eomes^−^CD90^+^RORγt^+^IL-22^+^ILC3. Data are representative of at least 2 independent experiments. (a-e, g, i) Data points are single mouse with mean. (a-c, e) Student’s t test, (d) Mann–Whitney test and (g, i) one-way ANOVA with Bonferroni correction. *p ≤ 0.05, **p ≤ 0.01, **** p ≤ 0.0001.

To understand how tissue immune responses were changed after GDAR2-2 colonization, we colonized ABX-treated mice with GDAR2-2 before infection with *C. rodentium* and measured cytokine secretion in colon explants 6 days post infection. In GDAR2-2-colonized mice, we found elevated colon IL-1β and IL-23 secretion (Figure 3(a-b)) alongside decreased IFN-γ secretion (Figure 3(c)). Similar IL-18 and TNF levels were detected in both groups (Figure 3(d-e)). IL-1β and IL-23 both induce type three innate lymphoid cell (ILC3) production of IL-22 which is required for protection from *C. rodentium* infection.^10,21,22^ To determine if GDAR2-2 induced ILC3 IL-22 secretion, we inoculated ABX-treated WT mice with or without GDAR2-2 and left untreated or infected with *C. rodentium* and performed flow cytometry of lamina propria cells. In the large intestine lamina propria of GDAR2-2-colonized mice, we found higher numbers of ILC3 with an increased proportion making IL-22 as compared to uncolonized mice (Figure 3(f-i)). After *C. rodentium* infection, we saw limited expansion of ILC3 or induction of IL-22 after infection in the absence of GDAR2-2 (Figure 3(f-i)). In contrast, in C. *rodentium*-infected GDAR2-2-colonized mice, we saw expansion of ILC3 with a higher number producing IL-22 (Figure 3(f-i)).

CD4^+^ T cell responses are also critical for *C. rodentium* clearance.^41,42^ However, we found no changes in total T cell numbers or in the frequency of T cell subsets after *C. rodentium* infection in GDAR2-2-colonized mice as compared to uncolonized controls (Figure S5(a-e)). To further confirm that protection after GDAR2-2 colonization was independent of adaptive immunity, we treated *Rag2^−/−^* mice with ABX and left uncolonized or colonized with GDAR2-2 before infection with *C. rodentium*. As in WT mice, GDAR2-2 colonization was sufficient to protect *Rag2^−/−^* mice from *C. rodentium* infection. Compared to uncolonized mice, *Rag2^−/−^* mice colonized with GDAR2-2 showed less weight loss and longer colon length than control *Rag2^−/–^*infected mice (Figure 5(f-g)), suggesting T cells were dispensable for GDAR2-2-induced protection against *C. rodentium* infection. Together, these data support that the expansion of IL-22^+^ILC3s is required for the GDAR2-2-induced protection,

### Colonic CX_3_CR1 MNPs promote barrier protection

**Figure 4.**
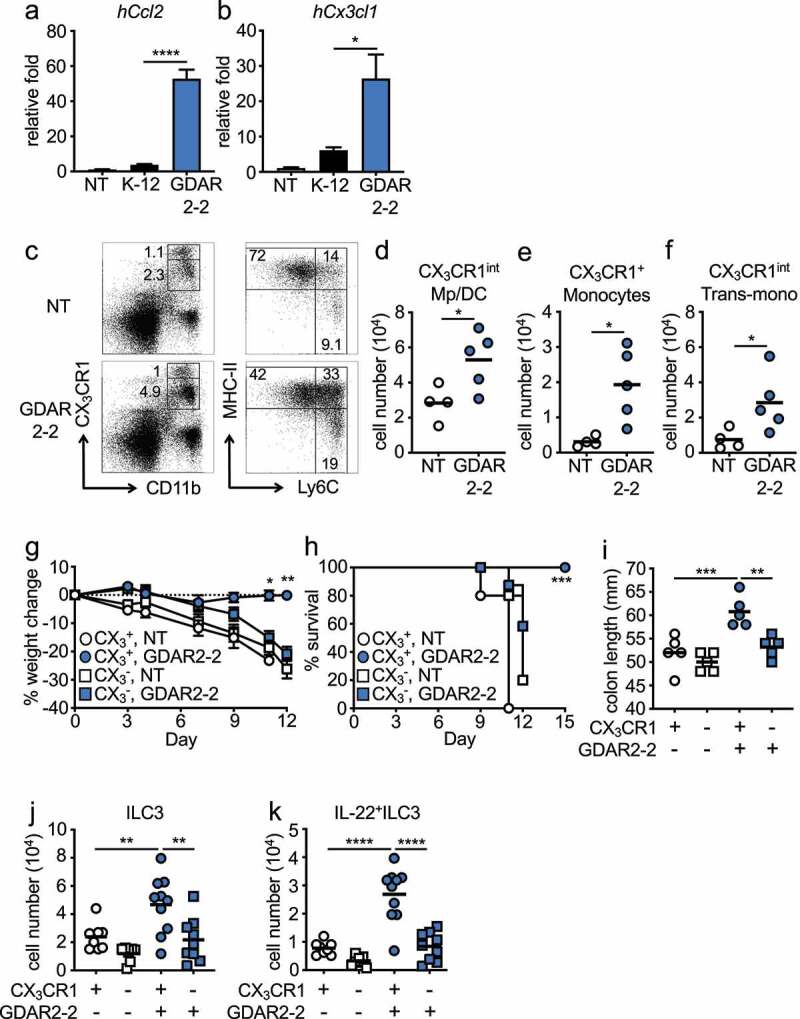
GDAR2-2 induced protection depends on CX_3_CR1^+^ MNPs. (a-b) Caco-2 cells were co-cultured with K-12, GDAR2-2 or left untreated. mRNA expression of (a) *hCcl2* and (b) *hCx3cl1* determined by qPCR are shown. (c-f) ABX-treated CX_3_CR1-GFP mice were colonized with GDAR2-2 or left un-colonized. 2 days later, CX_3_CR1^+^ MNPs from large intestine laminar propria were analyzed by flow cytometry. (c) Representative dot plots of live (left) and CD11b^+^CX_3_CR1^int^ population (right). Absolute numbers of (d) CX_3_CR1^int^ macrophages (Mp)/dendritic cells (DCs), (e) CX_3_CR1^+^ monocytes and (f) CX_3_CR1^int^ transitional (trans)-monocytes are shown. (g-k) DT-treated littermate control (CX_3_CR1+, CX_3_CR1^+^ MNP sufficient) and CX_3_CR1-DTR (CX_3_CR1-, CX_3_CR1^+^ MNP deficient) mice were treated with ABX and colonized with GDAR2-2 before infection with *C. rodentium*. (g) Weight change, (h) survival, (i) colon length 12 days post infection was shown. (j-k) Absolute number of (j) ILC3s and (k) IL-22^+^ILC3s 5 days post infection are shown. See also Figure S5. Data are representative of at least 2 independent experiments. (a-b) Data are shown as mean with SEM and K-12 and GDAR2-2 group are compared by student’s t test. (d-f) Data points are single mouse with mean and compared by Student’s t test. (g) Data are shown as mean ± SEM and compared by one-way ANOVA with Bonferroni correction for each time point. (h) log rank test. (i-k) Data points are single mouse with mean. One-way ANOVA with Bonferroni correction. *p ≤ 0.05, **p ≤ 0.01, *** p ≤ 0.001, **** p ≤ 0.0001.

Since we found increased mucosa colonization by GDAR2-2 as compared to K-12, we hypothesized that interactions with the epithelium could initiate the observed protective effects. To test if GDAR2-2 induced epithelial responses, we left untreated or co-cultured human intestinal cell line Caco-2 with K-12 or GDAR2-2 and measured gene expression by qPCR. We found increased expression of monocyte recruiting chemokines, *hCcl2, hCx3cl1, hCcl4, Ccl5* and *Ccl20*, after GDAR2-2 stimulation (Figure 4(a-b), Figure S6(a-c). Additionally, increased expressions of *hIl8* and pro-inflammatory cytokines such as *hIl23a, hIl6, hTnf, hIl1a, hIl1b* were found with no change in *hIl18* and *hIl1rn* expression after GDAR2-2 stimulation (Figure S6(d-k).

Previously, we demonstrated that CX_3_CR1^+^ MNPs are required to protect against *C. rodentium* infection through the induction of IL-22 production by ILC3s^10^. We hypothesized that the upregulated monocyte recruiting chemokines resulted in an influx of monocytes into the intestine that differentiated into CX_3_CR1^+^ MNPs to provide protection. In the intestine, CX_3_CR1^+^ MNPs can be divided into two major populations based on CX_3_CR1 expression, CX_3_CR1^hi^ and CX_3_CR1^int^ MNPs. CX_3_CR1^hi^ MNPs are considered macrophages^13,16,43^ whereas CX_3_CR1^int^ MNPs are composed of three subgroups including MHC-II^+^Ly6C^−^ cells that have characteristics of both macrophages and dendritic cells, MHC-II^−^Ly6C^+^ monocytes and the MHC-II^+^Ly6C^+^ transitional monocytes.^13,16,43^ To determine if GDAR2-2 regulated CX_3_CR1^+^ MNPs, we colonized ABX-treated mice with GDAR2-2 and analyzed MNP populations by flow cytometry. After GDAR2-2 colonization, we found expansion of CX_3_CR1^int^ macrophages and dendritic cells, CX_3_CR1^+^ monocytes and MHC-II^+^Ly6C^+^ transitional monocytes with no change in CX_3_CR1^hi^ macrophages in the intestine (Figure 4(c-f)). These data suggest that GDAR2-2-induced CX_3_CR1^+^ MNP expansion is likely driven by intestinal epithelium recruitment of monocytes.

To determine whether CX_3_CR1^+^ MNPs were critical for GDAR2-2-mediated protection, we utilized a mouse model where the diphtheria toxin receptor (DTR) is driven by the CX_3_CR1 promoter (CX_3_CR1-DTR)^10^ that allows for selective depletion of CX_3_CR1^+^ cells after diphtheria toxin (DT) administration.^12^ We administered DT to ABX-treated CX_3_CR1-DTR mice and WT littermate controls and colonized them with GDAR2-2. Two days later, we infected mice with *C. rodentium*. Colonization with GDAR2-2 did not protect mice from *C. rodentium* infection in the absence of CX_3_CR1^+^ MNPs. Similar to uncolonized mice, mice lacking CX_3_CR1^+^ MNPs showed increased weight loss, decreased survival, and decreased colon length compared to mice with normal CX_3_CR1^+^ MNPs (Figure 4(g-i)). In the steady state or after *C. rodentium* infection, in the absence of CX_3_CR1^+^ MNPs, GDAR2-2 induced neither colonic ILC3 expansion nor IL-22 secretion (Figure 4(j-k), Figure S7(a-b)). Together, these data indicate a critical role of CX_3_CR1^+^ MNPs in GDAR2-2-induced protection.

### Live mouse commensal E. coli promotes IL-1β secretion

**Figure 5.**
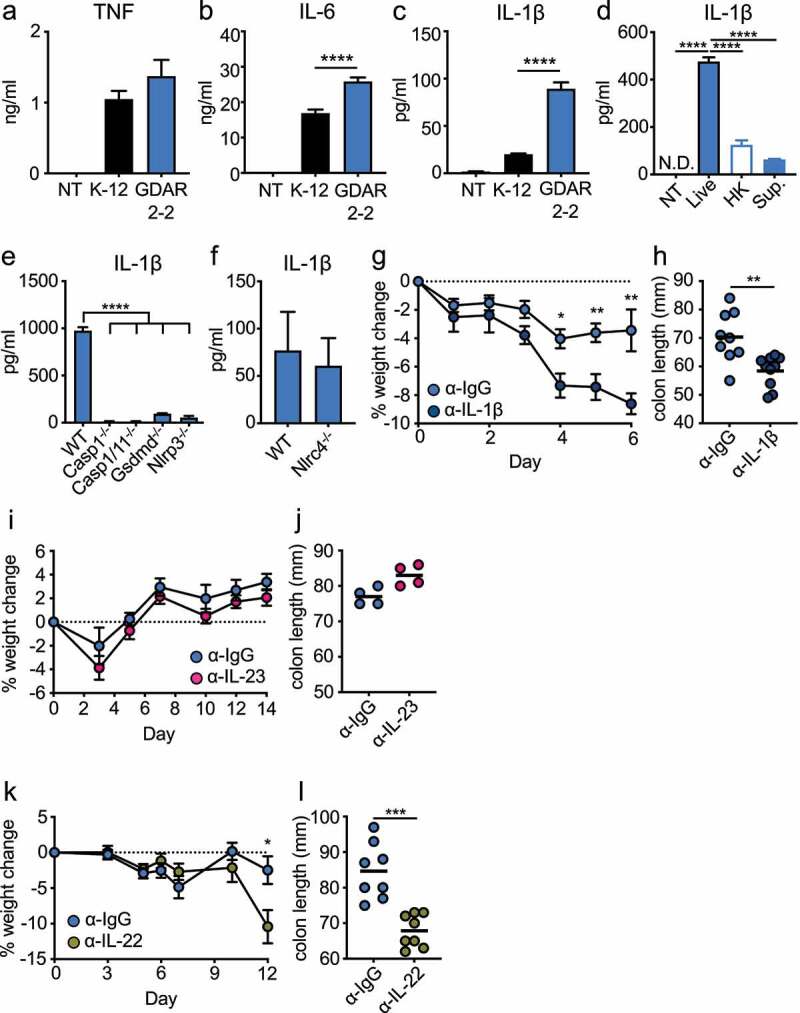
GDAR2-2-induced IL-1β secretion protects mice from *C. rodentium* infection. (a-c) WT bone marrow derived macrophages (BMDM) were co-cultured with K-12, GDAR2-2 or left untreated. Secretion of (a) IL-6, (b) TNF and (c) IL-1β were analyzed by LegendPlex. (d) Secretion of IL-1β from immortalized BMDM (iBMDM) co-cultured with live, heat-killed (HK), supernatant of GDAR2-2 or left untreated. (e-f) Secretion of IL-1β were detected from (e) WT, *casp11^−/−^, casp1/11^−/−^, Gsdmd^−/−^* and *Nlrp3^−/−^* iBMDM co-cultured with GDAR2-2 or from (f) WT and *Nlrc4^−/−^* BMDM co-cultured with GDAR2-2. (g-h) ABX-treated B6 mice were colonized with GDAR2-2, infected with *C. rodentium* and administered with α-IgG antibody (Ab) or α-IL-1β Ab. (g) Weight change and (h) colon length 6 days after infection were shown. (i-j) B6 mice were treated with ABX, colonized with GDAR2-2, infected with *C. rodentium* and treated with α-IgG Ab or α-IL-23 Ab. (i) Weight change and (j) colon length were shown. (k-l) ABX-treated B6 mice were colonized with GDAR2-2, infected with *C. rodentium* and treated with α-IgG Ab or α-IL-22 Ab. (k) Weight change and (l) colon length were shown. Data are pooled from (g,h,k,i) or are representative of (a-f, i, j) at least 2 independent experiments. (a-f) Data are shown as mean with SEM. (a-e) One-way ANOVA with Bonferroni correction. (f) Student’s t test. (g, i, k) Data are shown as mean ± SEM. Student’s t test at each time point. (h, j, l) Data points are single mouse with mean. Student’s t test. *p ≤ 0.05, **p ≤ 0.01, *** p ≤ 0.001, **** p ≤ 0.0001.

To determine if GDAR2-2 directly regulated macrophage cytokine secretion, we co-cultured WT bone marrow derived macrophages (BMDMs) with K-12 or GDAR2-2 and measured cytokine secretion by LegendPlex. As compared to K-12, co-culture with GDAR2-2 induced similar levels of TNF (Figure 5(a)) and undetectable IL-23 production (data not shown). In contrast, GDAR2-2 induced higher IL-6 and IL-1β secretion (Figure 5(b-c)). We next sought to understand if live GDAR2-2 or GDAR2-2 metabolites were required for IL-1β secretion. To test this, we co-cultured immortalized BMDMs (iBMDMs) with live GDAR2-2, heat-killed GDAR2-2, or GDAR2-2 culture supernatant and found that only live GDAR2-2 induced IL-1β production from iBMDMs (Figure 5(d)). To further understand if the inflammasome was required for GDAR2-2 induced IL-1β production, we utilized iBMDMs deficient for different inflammasome components and found that IL-1β production is abolished in *Casp11^−/−^, Casp1/11^−/−^, Gsdmd^−/−^* and *Nlrp3^−/−^* iBMDM (Figure 5(e)). However, IL-1β secretion is not blunted in *Nlrc4^−/−^* BMDM (Figure 5(f)). These data indicate that live GDAR2-2 is required to activate the inflammasome and results in IL-1β secretion from macrophages.

### IL-1β is required for GDAR2-2 protection against infection

Secretion of either IL-1β or IL-23 by CX_3_CR1^+^ MNPs can activate ILC3 production of IL-22.^10,44^ To determine whether IL-1β induced by GDAR2-2 was required for protection after *C. rodentium* infection, we administered isotype control or an antagonistic IL-1β antibody (Ab) to ABX-treated, GDAR2-2-colonized, and *C. rodentium*-infected WT mice. Mice treated with anti-IL-1β Ab showed significant weight loss and shorter colon length (Figure 5(g-h)), suggesting a critical role for IL-1β in GDAR2-2-mediated protection. We similarly used an antagonistic anti-IL-23 Ab. In contrast to IL-1β blockade, blocking IL-23 did not result in changes of weight loss or colon length (Figure 5(i-j)), suggesting that IL-23 is dispensable for GDAR2-2-induced protection. These results suggest that GDAR2-2-induced IL-1β secretion from CX_3_CR1^+^ MNPs is crucial for protection against *C. rodentium* infection.

To determine the role of IL-22 in GDAR2-2-induced protection, we similarly blocked IL-22 with an IL-22 blocking Ab in GDAR2-2 colonized *C. rodentium* infected mice. We found increased weight loss with shorter colon length in mice treated with anti-IL-22 blocking Ab as compared to isotype control, suggesting that GDAR2-2-induced IL-22 production is critical for protection from *C. rodentium* infection (Figure 5(k-l)).

### Select human commensal E. coli isolates promote barrier protection through IL-1β secretion

**Figure 6.**
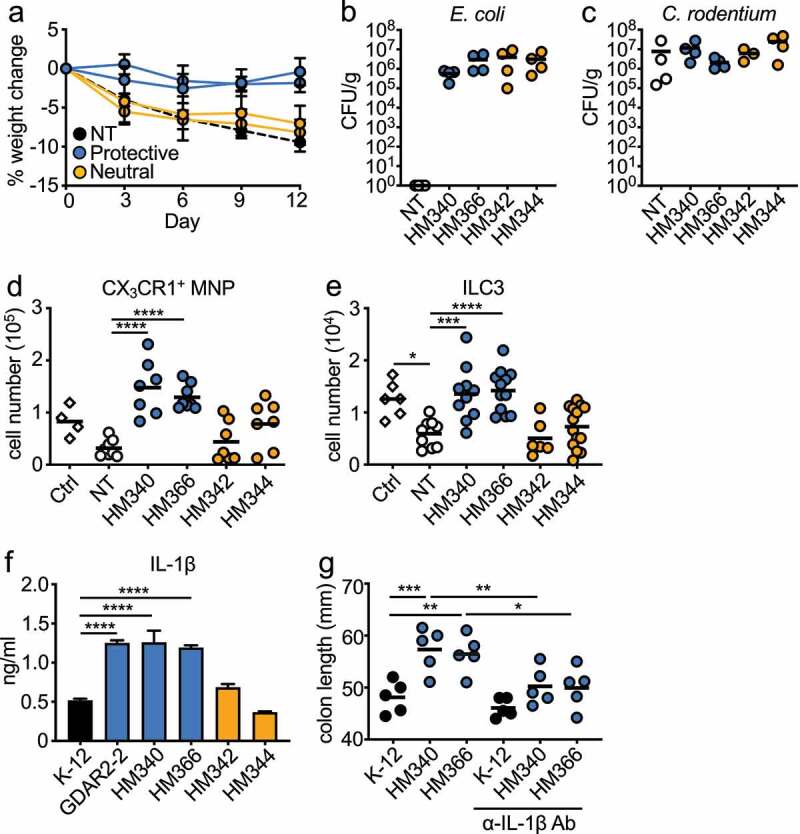
Select human *E. coli* isolates protect mice from *C. rodentium* infection through induction of IL-1β. (a-e) ABX-treated B6 mice were colonized with individual human *E. coli* isolates or left un-colonized and infected with *C. rodentium*. (a) Weight change. (b) *E. coli* and (c) *C. rodentium* titer in feces 5 days after infection analyzed by plating. Absolute number of (d) live CX_3_CR1^+^ MNPs and (e) lin^−^Eomes^−^CD90^+^RORγt^+^ILC3 of *C. rodentium* infected mice colonized with indicated *E. coli* isolate. (f) IL-1β secretion from iBMDM co-cultured with indicated *E. coli* isolate. (g) ABX-treated B6 mice were colonized with indicated *E. coli* isolates, infected with *C. rodentium* and administered with α-IgG Ab or α-IL-1β blocking Ab. Colon length is shown. Data are representative of at least 2 independent experiments. (a, f) Data are shown as mean ± SEM. (b-e, g) Data points are single mouse with mean. (d, e, g) One-way ANOVA with Bonferroni correction. *p ≤ 0.05, **p ≤ 0.01, *** p ≤ 0.001, **** p ≤ 0.0001.

In patients with inflammatory bowel diseases (IBD), increased levels of mucosa-associated bacteria such as *E. coli* are found.^7,8,45,46^ These microbes are thought to amplify pathology. To test if they could also regulate protection similar to our identified mouse *E. coli*, we assembled a panel of human microbiome project (HMP)^47^ mucosa-associated *E. coli* (Table 1). We treated mice with ABX as above before colonizing mice with individual human *E. coli* isolates. Mice were then infected with *C. rodentium*. While the majority did not protect from *C. rodentium*, we identified a subset of protective human *E. coli* as shown by reduced weight loss (Figure 6(a), Figure S8(a), Table 1). Similar to the mouse *E. coli*, GDAR2-2, we found comparable *E. coli* and *C. rodentium* fecal load in mice colonized with either protective or neutral *E. coli* isolates (Figure 6b-c). Supporting a similar mechanism of protection, we found increased colonic CX_3_CR1^+^ MNP and ILC3 numbers in mice colonized with protective as compared to neutral *E. coli* (Figure 6(d-e)).

**Table 1.** Characteristics of human *E. coli* isolates used in the study. Effect describes outcome in the *C. rodentium* infection model. HM339 is pathogenic when administered to mice in absence of C. rodentium infection. Crohn’s disease (CD) ulcerative colitis (UC), control = non IBD

Strain	ID	Isolation Site	IBD type	Accession No.	Effect
MS 85–1	HM337	Ileum	CD	ADWQ00000000	Protective
MS 119–7	HM339	Colon rectum	CD	ADWU00000000	Pathogenic
MS 124–1	HM340	Small bowel	CD	ADWT00000000	Protective
MS 145–7	HM341	Colon	CD	ADWS00000000	Neutral
MS 57–2	HM342	Ileum	Control	ADUG00000000	Neutral
MS 115–1	HM344	Colon	UC	ADTL00000000	Neutral
MS 16–3	HM345	Ileum	CD	ADUA00000000	Neutral
MS 69–1	HM347	Colon	Control	ADTP00000000	Neutral
MS 200–1	HM356	Ileum	Control	ADUC00000000	Neutral
MS 196–1	HM365	Colon	Control	ADUD00000000	Neutral
MS 198–1	HM366	Colon	Control	ADTJ00000000	Protective

To determine whether the protective human commensal *E. coli* also induced macrophage IL-1β production, we co-cultured individual human *E. coli* with iBMDM and analyzed supernatants for cytokine production by ELISA and LegendPlex. All *E. coli* induced similar levels of IL-6, TNF and IL-23 (Figure S8(c-e)). Similar to GDAR2-2, protective human *E. coli* induced higher IL-1β levels (Figure 6(f) and Figure S8(b)). To determine whether IL-1β induced by the protective human *E. coli* also contributed to protection from *C. rodentium*, we colonized ABX-treated mice with protective strains and treated them with isotype control or anti-IL-1β Ab. The protection was lost as shown by decreased colon length (Figure 6(g)) in the anti-IL-1β Ab-treated animals. These results indicate that, similar to the mouse commensal *E. coli* GDAR2-2, select human mucosa-adherent *E. coli* can protect against *C. rodentium*-induced colitis by enhancing macrophage IL-1β production.

## Discussion

Within the intestine, host-microbe interactions regulate many aspects of host physiology. We and others previously demonstrated that one of these functions is to promote barrier repair.^10,48,49^ However, the mechanisms by which individual bacterium and microbial signals regulate intestinal epithelial integrity remain unclear. Using antibiotic selection, we identified a multidrug resistant mouse commensal *E. coli*, GDAR2-2, that protects against *C. rodentium* infection and DSS-induced colitis in mice. We found that this protection is not due to direct competition with *C. rodentium* but depends on the regulation of host innate immune responses.

Colonization with GDAR2-2 induced IL-22^+^ILC3 expansion in mice. IL-22 is required for survival from *C. rodentium* infection^21^ by promoting intestinal epithelium proliferation and survival,^25^ including enhancing transit-amplifying cell proliferation,^32^ facilitating goblet cell regeneration and mucin secretion,^24^ and promoting anti-microbial peptide production.^21,26,50^ We found colonic upregulation of *Axin2* and *Ascl2*, markers of transit-amplifying cells, after GDAR2-2 colonization. We also found loss of protection when IL-22 was blocked in GDAR2-2-colonized mice, indicating its requirement for GDAR2-2-induced protection against *C. rodentium* infection.

Attachment of microbes to the gut epithelium is an important mechanism to regulate host immunity.^12,51^ In vivo, we found enriched GDAR2-2 at the intestinal epithelium, suggesting that protection might be downstream of epithelial attachment. Using the Caco-2 human colon cell line, we found up-regulation of monocyte chemoattractants upon GDAR2-2 stimulation. In line with this finding, GDAR2-2 colonization led to expansion of CX_3_CR1^+^ monocytes, CX_3_CR1^int^ transitional monocytes and CX_3_CR1^int^ macrophage/DC in the intestine. Previous studies showed that increased intestinal monocytes are pathogenic in colitis models^14,52,53^ while macrophages are found to be protective.^12,54,55^ Using mice that can be selectively depleted of CX_3_CR1^+^ cells,^12,13^ we demonstrated that the GDAR2-2-induced protection against *C. rodentium* infection is CX_3_CR1^+^ MNP dependent. We previously demonstrated that CX_3_CR1^+^ MNPs are required for ILC3 production of IL-22^10^. Depletion of CX_3_CR1^+^ MNPs also abolished GDAR2-2-induced IL-22^+^ILC3 expansion. Collectively, the commensal *E. coli* GDAR2-2 promotes barrier repair functions of CX_3_CR1^+^ MNPs that results in IL-22^+^ILC3 expansion.

Previous studies showed that secretion of IL-1β and IL-23 from CX_3_CR1^+^ MNPs activate ILC3s and induce IL-22 production from ILC3s.^10,23,44,56^ We found macrophage stimulation with live GDAR2-2 resulted in enhanced IL-1β secretion as compared to live K-12. In addition to activating IL-22 production, previous studies showed that IL-1β also supports Th17 cell responses with IL-1β required for early Th17 differentiation and synergizes with IL-6 and IL-23 to promote Th17 cell survival and effector functions.^57^ Accumulation of Th17 cells can lead to chronic inflammation.^58^ Although we found elevated IL-1β secretion from GDAR2-2 stimulated macrophages, we did not see expansion of Th17 cells nor other T cell populations. Further, neutralization of IL-1β but not IL-23 abrogated the protective effect of GDAR2-2 in ABX-treated, GDAR2-2-colonized mice. These findings indicate the importance of IL-1β activation of IL-22 production by ILC3 in the GDAR2-2-induced protection from *C. rodentium* infection.

In IBD patients, a shift in the microbiota composition has been identified with increased mucosa-associated bacteria including *Proteobacteria*.^7,8,45,46^ Using human *E. coli* isolated as part of the NIH human microbiome project^47^ we identified several human *E. coli* isolates that also protect from *C. rodentium* infection. Similar to GDAR2-2, these *E.coli* isolates increase intestinal ILC3 numbers and induce macrophage IL-1β secretion. As with GDAR2-2, blocking IL-1β ameliorates their protection. Although increased *Proteobacteria* is associated with IBD and certain human *E. coli* isolates were isolated from IBD patients, these results highlight that distinct commensals can play distinct roles depending on tissue context.

IL-1β is a pro-inflammatory cytokine that promotes various inflammatory disorders, including rheumatoid arthritis (RA) and type 2 diabetes (T2D) where neutralization of IL-1β effectively represses inflammation and reduces disease severity.^59,60^ Similar to RA and T2D, increased IL-1β is found in IBD patients.^61,62^ IL-1β can increase intestinal tight junction permeability,^63–65^ which can increase intestinal bacteria penetration from the lumen to lamina propria and results in colitis in mouse models.^66^ However, blocking IL-1β does not ameliorate symptoms in IBD patients.^61,67^ Protective effects of IL-1β are found in the skin where bacteria induced IL-1β secretion results in skin regeneration after injury.^68^ Further, additional studies support an anti-inflammatory role for IL-1β which promotes Treg functions and induces IgA production.^69–71^

Work by Seo et al. shows that *Proteus mirabilis* activates monocyte secretion of IL-1β.^52^ Unlike GDAR2-2, *P. mirabilis* induction of IL-1β is pathogenic in DSS-induced colitis in WT mice. *P. mirabilis* is a pathobiont^52,72^ that carries virulence factors such as Proteus toxic agglutinin and type III secretion systems.^73^ In addition, Viladomiu and Metz et al. demonstrate that the large subunit of propanediol dehydratase (PduC) encoded by Adherent-invasive *E. coli* (AIEC) triggers IL-1β secretion from CX_3_CR1^+^ MNPs.^56^ Similar to *P. mirabilis*, IL-1β induced by PduC-encoding AIEC also causes severe pathology in DSS-induced colitis in *Il10*-deficient mice. The metabolic activity of PduC also exacerbates T cell-dependent colitis.^56^ On its own, GDAR2-2 does not induce intestinal pathology and these data highlight that immune activation in combination with microbial characteristics together contribute to the disease state of the intestine.

In addition to the ability of IL-1β to induce IL-22 secretion by ILC3, a recent study demonstrated that IL-1-signaling on mesenchymal cells results in increased production of Wnt agonists that promote intestinal stem cell proliferation and can protect in DSS-induced colitis and after *C. rodentium* infection.^33^ However, we did not see up-regulation of Wnt agonist, Rspo3, and the Wnt target stem cell gene, Lgr5, suggesting that GDAR2-2 does not protect mice from *C. rodentium* infection through Wnt signaling to induce intestinal stem cell regeneration. Instead, we found up-regulation of *Axin2* and *Ascl2*, which are the transit-amplifying cell markers induced by IL-22 signaling, suggesting that GDAR2-2 provides protection likely through IL-22-induced transit-amplifying cell proliferation. The loss of protection in the GDAR2-2-colonized mice given anti-IL-22 blocking Ab also strengthens this finding. Together, these results support the protective role of GDAR2-2 against *C. rodentium* infection through inducing barrier repair by activating the IL-1β-IL-22 pathway. This may indicate spatial interactions between CX_3_CR1^+^ MNPs and ILC3s instead of activating mesenchymal cells that secret Wnt agonist after GDAR2-2 colonization to protect from C. rodentium infection.

As microbiota composition changes are often found in IBD and autoimmune diseases,^9^ understanding how individual commensal bacterium shapes the host immune responses will offer important clues for developing strategies to ameliorate disease. Here we describe the identification of a subset of intestinal mucosa-associated *E. coli* that induce CX_3_CR1^+^ MNP IL-1β production to protect the intestinal barrier. Together, these findings provide potential therapeutic interventions for IBD by regulating microbial composition and signals as well as revisiting the role of IL-β.

## Materials and methods

### Mice

CX_3_CR1-DTR mice were previously described^10,12^ . C57BL/6 J (JAX # 000664) and CX_3_CR1-GFP (JAX # 005582) mice are from Jackson Laboratories and Rag2^−/−^ (RAGN12) are from Taconic. All mice are bred in-house under standard SPF conditions at the animal facilities of Baylor College of Medicine or Memorial Sloan Kettering Cancer Center. All mouse experiments were performed with mice between 8 and 12 weeks of age with males and females at similar ratios, unless otherwise specified. Littermate controls were used for each experiment and mice were randomly assigned to experimental groups with a minimum of 3 mice per group. All experiments were performed in accordance with approved protocols by the Institutional Animal Care and Usage Committee at Baylor College of Medicine or Memorial Sloan Kettering Cancer Center.

### Depletion of gut microbiota

Animals were provided with 1 g/L ampicillin (A; Sigma, A0166), 500 mg/L vancomycin (V; Sigma, V2002), 1 g/L neomycin sulfate (N; Sigma, N1876), and 1 g/L metronidazole (M; Sigma, M3761) in drinking water for four weeks^4,13^ or treated with single dose of 20 mg/ml streptomycin (Sigma, S9137-25 G) in 100 μl water by per os (P.O.) and with 1 g/1 L ampicillin (Fisher BioReagent, BP176025) in drinking water for 1–2 weeks. Ampicillin water was replaced with regular drinking water 2 days before *E. coli* colonization as described below. Microbiota depletion was confirmed by bacterial DNA in feces as detected by real time qPCR.

### GDAR2-2 isolation and sequencing

Fecal pellets from AVMN-treated mice were resuspended in PBS to 100 mg/ml and dilutions were plated on blood agar plates (Fisher) and cultured overnight at 37°C under normal or anaerobic conditions using BD GasPak (Fisher). For sequencing, genomic DNA was extracted with phenol chloroform, and DNA was sheared to 15kb using Covaris g-TUBE® devices, allowing for sizes 5 kb and larger. The library preparation was carried out using SMRTbell Template kit 1.0 Exo VII protocol and the sample was barcoded with PacBio Adaptor. Genome sequencing was performed using the Pacific Biosciences Sequel sequencing platform. Long reads were assembled de novo into two contigs (main chromosome and 1 plasmids) using Canu (v. 1.6).^73^ Gene prediction and annotation were carried out using the webservice PATRIC.^74^ Genomic visualization was performed using Circos v0.69–9.^75^ Genomic comparison was done using the PATRIC webservice and phylogenomic reconstructions were done using the GToTree pipeline and its associated dependencies.^76–80^ Sequencing reads and the genome assembly were submitted to NCBI under the bioproject PRJNA725420.

### Bacteria

K-12 (ATCC® PTA-7555) and *Citrobacter rodentium* (ATCC® 51,459) are from ATCC. Human Microbiome Project^46^ *Escherichia coli* isolates are from ATCC with details in Table 1. For heat-killed GDAR2-2, GDAR2-2 was grown to OD_600_ = 1 and was left at 85°C for 15 min. For GDAR GDAR2-2 supernatant, GDAR2-2 was grown to OD_600_ = 1, spun down at 3000 g, RT for 10 min. The supernatant was then filtered through a 0.22 μm filter.

### Colonization of mice with E. coli

Mice were treated with ABX to disrupt microbiota before colonizing with *E. coli. E. coli* was incubated at 37°C overnight with shaking at 220 rpm in LB broth. ABX-treated animals were colonized with *E. coli* isolates: K-12, GDAR2-2 or HMP *E. coli* at 1 × 10^8^ colony forming unit (CFU) in 100 μl PBS. Colonization was confirmed by real time qPCR with *E. coli*-specific qPCR primers before infecting with *C. rodentium* as described below.

### C. rodentium infection

ABX-treated mice were left uncolonized or colonized with *E. coli* 2 days before *C. rodentium* infection as described above. *C. rodentium* was grown overnight in LB broth at 37°C with shaking at 220 rpm. Stationary phase *C. rodentium* was diluted 1:100 in LB broth and grown at 37°C with shaking until log phase. Mice were infected with 1 × 10^10^ colony forming units (CFU) of *C. rodentium* in 100 μl PBS. Infection was confirmed by real time qPCR with *C. rodentium*-specific qPCR primers.

### DSS-induced colitis

Mice were given 2% dextran sodium sulfate (DSS) (Alfa Aesar, J63606) in drinking water for 5 days after which they were switched to regular drinking water.

### Diphtheria toxin (DT) and blocking Ab administration

Starting 2 days before *E. coli* colonization, mice were intraperitoneally (I.P.) injected every other day with 200ng DT (Sigma, D0564) in PBS. For IL-1β blocking, mice were injected I.P. with 200 μg anti-IgG control Ab (BioCell, BE0290) or anti-IL-1β blocking Ab (BioCell, BE0246) a day before *E. coli* inoculation. Mice were then injected i.p. every other day starting at day of *E. coli* colonization with 50 μg anti-IgG control Ab or anti-IL-1β blocking Ab. For IL-23 blocking experiment, mice were injected twice a week by i.p. with 50 μg anti-IgG control Ab (BioCell, BE0290) or anti-IL-23 blocking Ab (BioCell, BE0313) starting from 2 days before *E. coli* colonization. For IL-22 blocking, mice were i.p. injected with 100 μg anti-IgG control Ab or anti-IL-22 blocking Ab (ThermoFisher, 16–7222-38) every other day from the day of *E. coli* inoculation.

### Fecal albumin detection

Feces were freshly collected at day 4 after *C. rodentium* infection from ABX-treated mice colonized with K-12, GDAR2-2 or left untreated. Feces albumin was detected by Bromocresol Green Albumin Assay Kit (Sigma-Aldrich, MAK124).

### Colon explant

Colon explant was performed as described elsewhere.^81^ In brief, 6 days after *C. rodentium* infection, same area of the colons were isolated from each mouse, opened and washed. Colons were homogenized in lysis buffer supplemented with protease inhibitor. After centrifuging down the debris, supernatant was collected for cytokine detection using a customized LegendPlex kit (BioLegend).

### Lamina propria cell isolation

Isolation of cells from large intestine laminar propria has been described previously.^13^ Briefly, large intestines were opened longitudinally and washed in cold PBS to remove luminal contents. Tissue was cut into 1 cm sections and incubated while shaking in PBS with 1 mM DTT (Sigma, D9779), 30 mM EDTA (ThermoFisher, AM9621) and 10 mM HEPES (HyClone, SH30237.01) for 10 minutes at 37°C followed by PBS with 30 mM EDTA and 10 mM HEPES for 10 minutes at 37°C. Tissue was then digested at 37°C in a humidified chamber in RPMI/10% FBS with 200 U/ml type 8 collagenase (Sigma, C2139) and 150ug/ml DnaseI (Sigma, DN25) followed by separation on a 40% /80% Percoll (GE Health, 17–0891-01) gradient.

### Antibody, staining, and flow cytometry

Flow cytometry analysis was performed with BD LSRII or Cytek Aurora and analyzed with FlowJo (Tree Star Inc.). Antibodies were from BD, eBiosciences, and BioLegend. For live/dead cell staining, DAPI (Sigma, D9542) or live/dead fixable blue dead cell stain (ThermoFisher, L34962) were used. To prevent nonspecific binding, CD16/32 Ab were used on all samples. For cell counts, 123 count ebeads (ThermoFisher, 01–1234) were used to determine absolute cell numbers. For cytokine staining, cells were activated with 50 ng/ml phorbol myristate acetate and 1 μM ionomycin for 4 hours. Together with intracellular staining, cells were fixed, permeabilized and stained with anti-mouse IL-22, T-bet, GATA-3, RORγt, and Foxp3 according to the Foxp3/Transcription Factor Staining Buffer Set instructions (ThermoFisher, 00–5523-00). For ILCs, lineage (CD3, CD5, CD8, Ly6G, CD19) negative (lin^−^) CD90^+^ cells were analyzed for transcription factors and IL-22. For CX_3_CR1^+^ MNP staining, CX_3_CR1-GFP, CD11b, MHC-II and Ly6C were used. For T cell staining, CD3, TCR-β and CD4 were used. Surface staining was stained in FACS buffer (PBS with 2 mM EDTA and 2% FBS) for 15 min, 4°C in the dark. All antibody information is listed below.

**Table:** 

Antibody	Fluorochrome	Clone	Source	Identifier
Anti-mouse CD3	APC-eflour 780	145–2C11	BioLegend	100,330
Anti-mouse CD3	FITC	17A2	BioLegend	100,204
Anti-mouse CD5	FITC	53–7.3	ThermoFisher	11–0051-85
Anti-mouse CD4	Alexa fluor 700	RM4-5	ThermoFisher	56–0042-82
Anti-mouse CD4	FITC	RM4-5	BioLegend	100,510
Anti-mouse CD8a	FITC	53–6.7	ThermoFisher	11–0081-85
Anti-mouse CD11b	APC/Cy7	M1/70	BioLegend	101,266
Anti-mouse 16/32	-	93	BioLegend	101,302
Anti-mouse CD19	FITC	MB19-1	ThermoFisher	11–0191-85
Anti-mouse CD90.2	BUV395	53–2.1	BD	565257
Anti-mouse Eomus	PE-efluor 610	Dan11mag	ThermoFisher	61–4875-82
Anti-mouse Foxp3	APC	FJK-16s	ThermoFisher	50–5773-82
Anti-mouse GATA-3	BV421	16E10A23	BioLegend	653,814
Anti-mouse IL-22	APC	IL22J0P	ThermoFisher	17–7222-82
Anti-mouse Ly6C	BV510	HK1.4	BioLegend	128,033
Anti-mouse Ly6G	FITC	1A8	BD	551460
Anti-mouse IA/IE	Pacific Blue	M5/114.15.2	BioLegend	107,620
Anti-mouse RORγt	PE	B2D	ThermoFisher	12–6981-82
Anti-mouse T-bet	PE/Cy7	4B10	ThermoFisher	25–5825-82
Anti-mouse TCR-β	PerCP/Cy5.5	H57-597	BioLegend	109,228
CX_3_CR1 reporter	eGFP	-	-	-

### Bacterial DNA isolation

Bacterial DNA was isolated from feces or intestines using DNeasy PowerSoil Kit (QIAGEN, 12,888–100) according to manufacturer’s protocol.

### RNA extraction and real time RT-PCR

RNA extraction from Caco-2 cells were performed with Trizol (ThermoFisher, 15,596,018) following manufacturer’s protocol. RNA was reverse transcribed to cDNA using iScript Reverse Transcription Supermix (BIO-RAD, 1,708,841). qPCR was performed with iTaq Universal SYBR Green Supermix (BIO-RAD, 1,725,125), 5 μmol of forward and reverse primers listed below, 100 ng cDNA using a QuantStudio 6 Pro (ThermoFisher Scientific). The qPCR program was 95°C for 2 min to initiate the reaction followed by 95°C for 15s, 60°C for 30s, and 72°C for 30s for a total of 40 cycles. Relative expression of target genes expression was determined by calculating delta Ct compare to reference gene.

**Table:** 

Primer	Sequence
Pan 16s-F	CGGTGAATACGTTCYCGG
Pan 16s-R	GGWTACCTTGTTACGACTT
E. coli 23s-F	GGTAGAGCACTGTTTTGGCA
E. coli 23s-R	TGTCTCCCGTGATAACTTTCTC
C. rodentium-F	ATGCCGCAGATGAGACAGTTG
C. rodentium-R	CGTCAGCAGCCTTTTCAGCTA
Human Actb-F	CTACCTTCAACTCCATCATGAAGTG
Human Actb-R	TGCGCTCAGGAGGAGC
Human Ccl2-F	GCCTCCAGCATGAAAGTCTC
Human Ccl2-R	AGGTGACTGGGGCATTGAT
Human Ccl4-F	CAGCCAGCTGTGGTATTCCAA
Human Ccl4-R	CTCCTGGACCCAGGATTCACT
Human Ccl5-F	ACACCCTGCTGCTTTGCCTACA
Human Ccl5-R	TCCCGAACCCATTTCTTCTCTG
Human Ccl20-F	CCCAAAGAACTGGGTACTCAAC
Human Ccl20-R	TCCAAGACAGCAGTCAAAGTTG
Human Cx3cl1-F	TGGCTGCTCCGCTTGGC
Human Cx3cl1-R	CCTGGTTCTGTTGATAGTGGATGAG
Human Il1α-F	TGTATGTGACTGCCCAAGATGAAG
Human Il1α-R	AGAGGAGGTTGGTCTCACTACC
Human Il1β-F	CCACAGACCTTCCAGGAGAAT
Human Il1β-R	GTGCAGTTCAGTGATCGTACAGG
Human Il1rn-F	CAGCTGGAGGCAGTTAACATCAC
Human Il1rn-R	CCACTGTCTGAGCGGATGAA
Human Il6-F	AGACAGCCACTCACCTCTTCAG
Human Il6-R	TTCTGCCAGTGCCTCTTTGCTG
Human Il8-F	TGGCTCTCTTGGCAGCCTTC
Human Il8-R	TGCACCCAGTTTTCCTTGGG
Human Il18-F	CCTGCTGCAGTCTACACAGC
Human Il18-R	CAGCCATCTTTATTCCTGCGAC
Human Il23a-F	TGGAGTGCACATCCACTAGTG
Human Il23a-R	CCTTTGCAAGCAGAACTGACT
Human Tnf-F	GTAGCCCATGTTGTAGCAAACC
Human Tnf-R	AGAGGACCTGGGAGTAGATGAG
Mouse Ascl2-F	CTACTCGTCGGAGGAAAG
Mouse Ascl2-R	ACTAGACAGCATGGGTAAG
Mouse Axin2-F	GAGTGGACGTGTGCCGACCTCA
Mouse Axin2-R	GGTGGCTGGTGCAAAGACATAG
Mouse Cldn1-F	CTGGAAGATGATGAGGTGCAGAAGA
Mouse Cldn1-R	CCACTAATGTCGCCAGACCTGAA
Mouse Lgr5-F	GACAATGCTCTCACAGAC
Mouse Lgr5-R	GGAGTGGATTCTATTATTATGG
Mouse Rspo3-F	TTGACAGTTGCCCAGAAGGG
Mouse Rspo3-R	CTGGCCTCACAGTGTACAATACT

### Cell lines and micorbial co-culture

For Caco-2 cells (ATCC® HTB-37), 2.5 × 10^4^ cells were seeded in 24-well tissue culture (TC) plate in 1 ml DMEM (Corning, MT10017CV) supplemented with 10% FBS (Gibco, 26,140–079), 10 mM HEPES (HyClone, SH30237.01), 2 mM glutamine (HyClone, SH30034.01), 1 mM sodium pyruvate (HyClone, SH30239.01), 1X MEM non-essential amino acids (ThermoFisher, 11,140–050), 100 unit/ml penicillin-streptomycin (HyClone, SV30010). Media was changed every other day until cells were confluent. For coculture with Caco-2 cells, one day prior to *E. coli* co-culture, cells were washed twice with PBS and media was replaced with complete DMEM without antibiotics. The next day, cells were spin infected at RT, 1000 x g for 10 min and co-cultured with indicated *E. coli* at MOI = 20 for 3 hours. Cells were washed once with PBS and resuspend in Trizol for RNA extraction. Immortalized bone marrow derived macrophages (iBMDM) were gifts from Dr. Jonathan C. Kagan.^82^ 2 × 10^5^ iBMDM cells were seeded in 96-well TC plate in 200 μl complete DMEM without antibiotics and incubated overnight. iBMDM cells were then spin infected at RT, 1000 x g for 10 min with indicated *E. coli* at MOI = 20 and incubated for 10 min. Cells were washed twice with sterile PBS and media was replaced with complete DMEM containing 100 μg/ml gentamycin (Sigma, G1272). After 1 hour incubation, cells were washed once with PBS, and media was replaced with complete DMEM with 20 μg/ml gentamycin. After 10 or 24 hour incubation, supernatant was collected for cytokine detection by IL-1β ELISA kit (ThermoFisher, 88–7013-88) or customized LegendPlex kit (BioLegend).

### Bone marrow derived macrophage (BMDM) culture

Femurs and tibias from WT and *Nlrc4^−/−^* (from Dr Isabella Rauch) mice were used for BMDM culture. BM was isolated from the indicated mice, treated with RBC lysis buffer and plated at a density of 1x10^6^/ml in a 15 cm TC dish in bone marrow macrophage (BMM) medium (complete DMEM supplemented with 30% L-929 cell-conditioned medium). At day 3, same amount of the BMM media was added to the cell culture. Day 6 cell culture was harvested for microbial stimulation as described for the iBMDM.

### Histopathology

Tissues were fixed in 10% buffered neutral buffered formalin (Fisher Scientific, SF100-4). Fixed tissues were paraffin embedded, processed, and stained with hematoxylin and eosin (H&E) using standard protocols. Samples were scored from 0 to 4 using established criteria^12^- Grade 0: Histology is normal. Grade 1: Mild inflammatory infiltrate in submucosa with edema. Minor erosion and muscularis remains intact. Grade 2: Grade 1 histology appear over 50% of the tissues. Grade 3: Severe inflammatory infiltration (predominantly neutrophil infiltrate) with edema. Ulceration in submucosa to muscularis layer. Grade 4: Grade3 histology shows in over 50% of the tissues.

### Statistics

Normality was determined by Shapiro Wilk test. If the data was normally distributed, for two groups, Student’s t test was used; for more than two groups, one-way ANOVA with Bonferroni correction was used. If the data was not normally distributed, for two groups, Mann–Whitney test was used; for more than 2 groups, Kruskal–Wallis test with Dunn’s multiple comparison was used. For survival, log rank test was used. All statistics are un-paired, 2-tailed with 95% confidence interval. Data are shown in mean or mean ± SEM. Significance is determined as ns:p > 0.05, *p ≤ 0.05, **p ≤ 0.01, *** p ≤ 0.001, **** p ≤ 0.0001. Data was plotted with GraphPad Prism 8.4.3.

## Supplementary Material

Supplemental Material

## Data Availability

Sequencing reads and the genome assembly are openly available in NCBI under bioproject PRJNA725420
