# Supplementary material for: Interleukin-1β secretion induced by mucosa-associated gut commensal bacteria promotes intestinal barrier repair
Source: Gut Microbes. 2022 Jan 6;14(1):2014772. doi: 10.1080/19490976.2021.2014772 (PMC8741296; doi:10.1080/19490976.2021.2014772)
Supplement: Supplemental Material [file KGMI_A_2014772_SM3178.pdf]

a

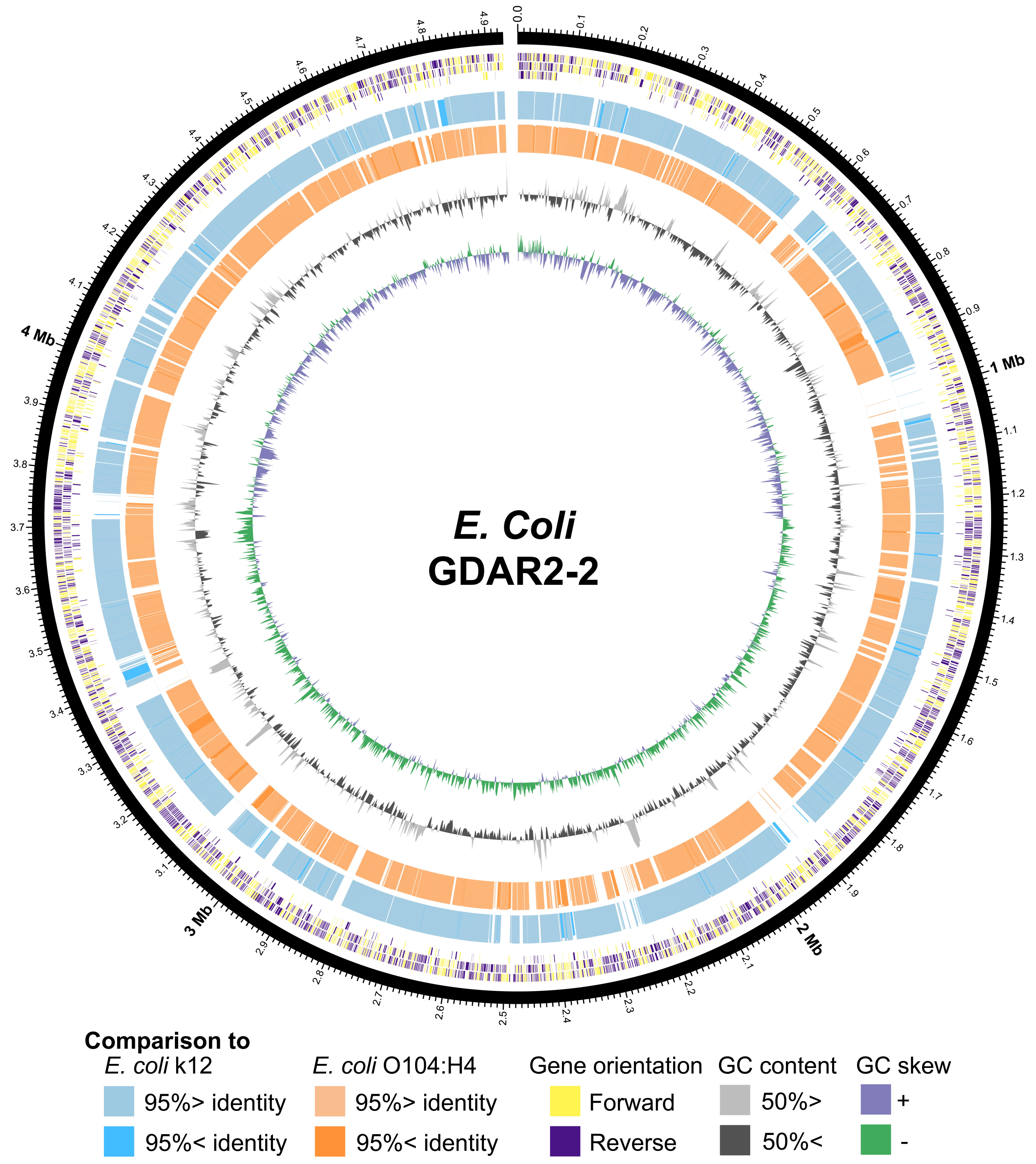

Supplementary Figure 1. Circular genome map of *E. coli* isolate GDAR2-2. The genome of the GDAR2-2 isolate was sequenced using PacBio and assembled *de novo* into 2 contigs including 1 main chromosome and 1 plasmid. The complete circular genome was 4,928,781 bp in length with a GC content of 49.34% and encoded 4,854 ORFs. The genome of GDAR2-2 was annotated using PATRIC and visualized with Circos. Comparative genomic analysis was performed for GDAR2-2 genome (black outer ring) using K-12 *E. coli* (blue ring) and closely related human *E. coli* 104:H4 (GCF_002209105.2, red ring) as reference genomes.

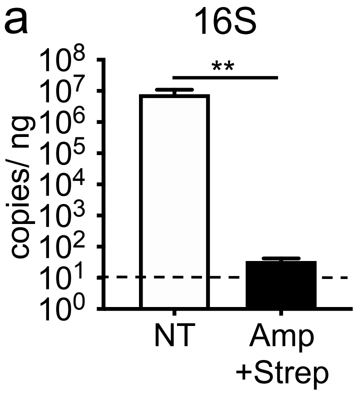

Supplementary Figure 2. Antibiotics treatment disrupts intestinal microbiota. WT C57BL/6 (B6) mice were treated with ampicillin and streptomycin or left untreated for 1-2 weeks. Feces were collected and analyzed for pan 16s rRNA expression by qPCR. Dotted line represents limit of detection for assay. Data are representative of at least 2 independent experiments. Data are shown as mean ± SEM. Mann-Whitney test was used. $^{**}p \leq 0.01$.

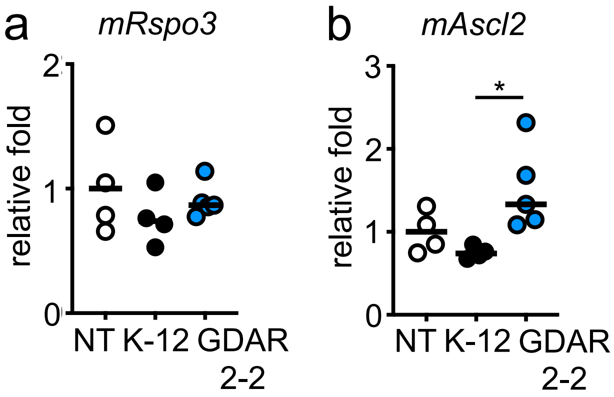

Supplementary Figure 3. Mouse commensal *E. coli* promotes transit-amplifying cell proliferation. ABX-treated WT B6 mice were colonized with K-12, GDAR2-2 or left un-colonized and infected with *C. rodentium*. 4 days after infection, colon expression of (a) mRspo3 and (b) mAscl2 was measured by qPCR. Data are representative of at least 2 independent experiments. (a,b) Data are shown in mean and compared by one-way ANOVA with Bonferroni correction. $^*p \leq 0.05$.

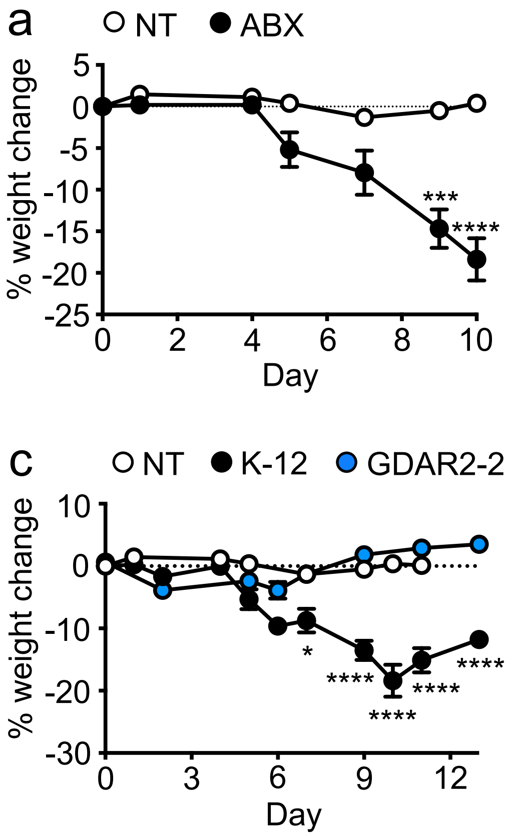

Supplementary Figure 4. Mouse commensal E. coli protects mice from DSS-induced colitis. (a-b) WT C57BL/6 (B6) mice were treated with ABX or left untreated before being given 2% DSS in drinking water. (a) Weight change and (b) survival are shown. (c-d) B6 mice without ABX treatment or treated with ABX and colonized with K-12 or GDAR2-2 were treated with 2% DSS. (c) Weight change and (d) survival are shown. Data are representative of at least 2 independent experiments. (a, c) Data are shown as mean ± SEM. (a) Student's t test or (c) one-way ANOVA with Bonferroni correction for each time point. (b, d) log rank test. *$p \leq 0.05$, **$p \leq 0.01$, *** $p \leq 0.001$, **** $p \leq 0.0001$.

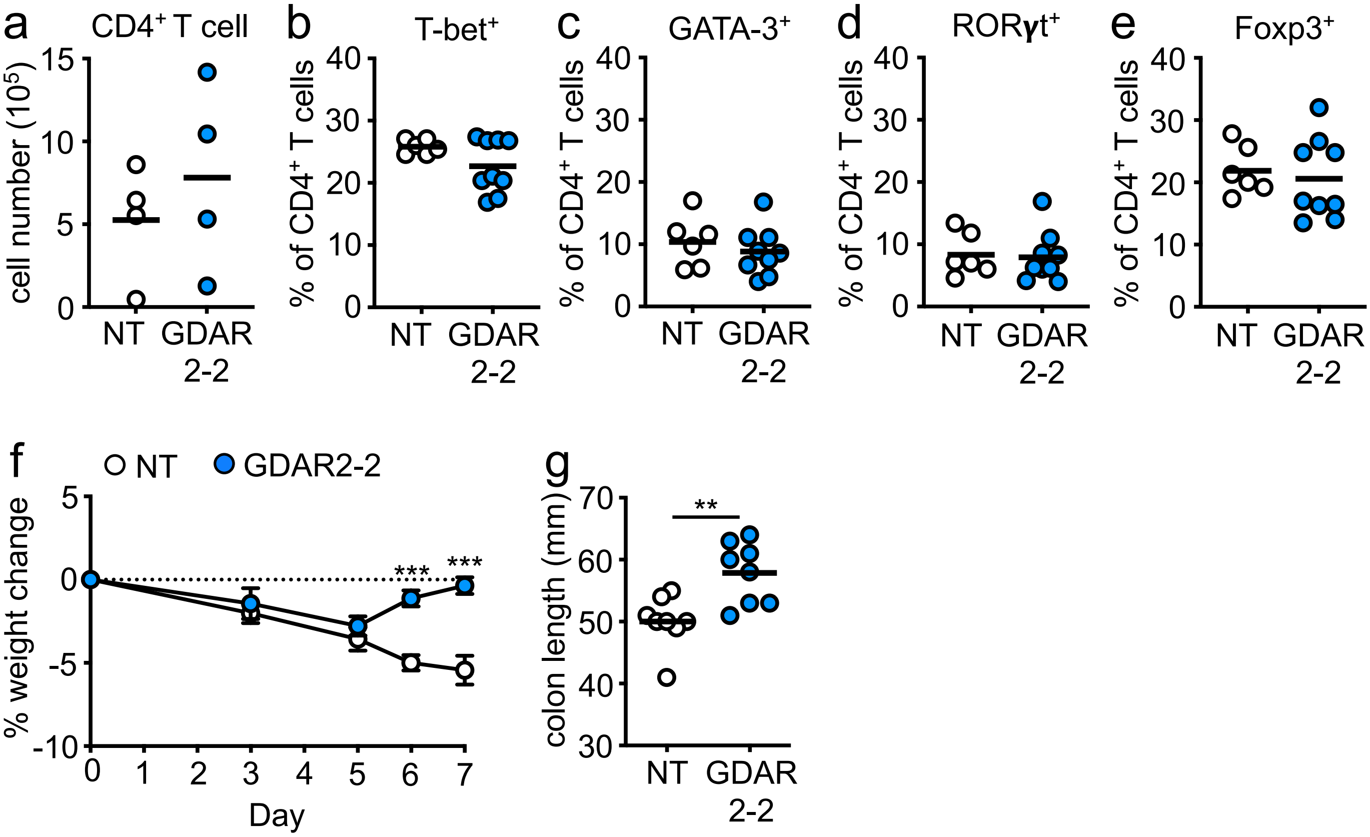

Supplementary Figure 5. *E. coli* GDAR2-2 protects mice from *C. rodentium* in a T cell-independent manner. (a-e) ABX-treated B6 mice with or without GDAR2-2 colonization were infected with *C. rodentium* and intestinal cell populations were analyzed by flow cytometry. (a) Absolute number of large intestine $CD4^+$ T cells (live $CD3^+TCR\text{-}\beta^+CD4^+$) Percentage of (b) T-bet$^+$, (c) GATA-3$^+$, (d) RORγt$^+$ and (e) Foxp3$^+$ of $CD4^+$ cells are shown. (f-g) ABX-treated *Rag2*$^{-/-}$ mice were colonized with or without GDAR2-2 and infected with *C. rodentium*. (f) Weight change and (g) colon length are shown. Data are representative of at least 2 independent experiments. (a-e, g) Data points are single mouse with mean compared by Student's t test. (f) Data are shown as mean ± SEM and compared with Student's t test for each time point. $^{**}p \leq 0.01$, $^{***}p \leq 0.001$.

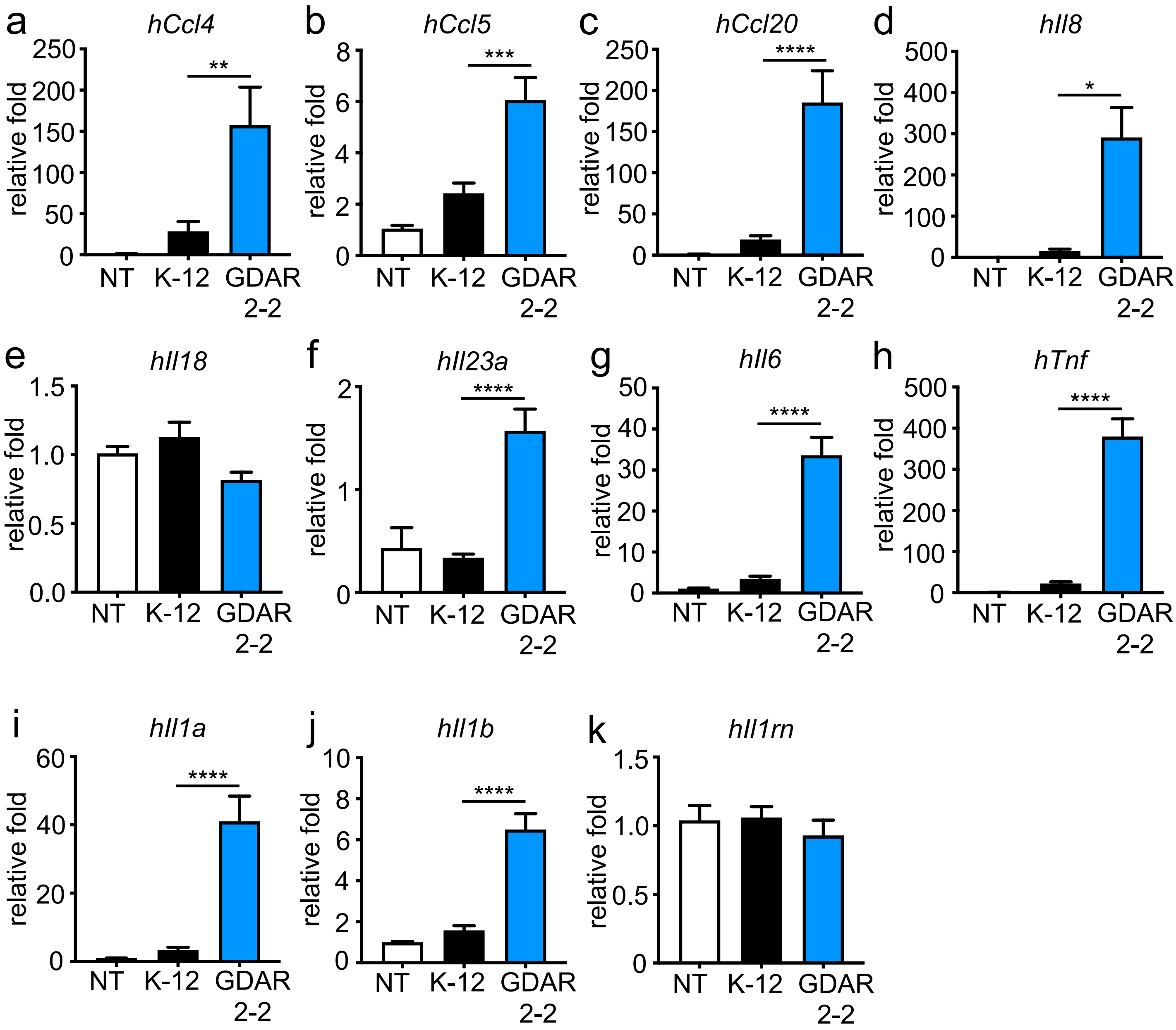

Supplementary Figure 6. GDAR2-2 induces monocyte chemoattractant in Caco-2 cells. Caco-2 cells were co-cultured with K-12, GDAR2-2 or left untreated for 3 hours. Expression of (a) *hCcl4*, (b) *hCcl5*, (c) *hCcl20*, (d) *hIl8*, (e) *hIl18* (f) *hIl23a*, (g) *hIl6*, (h) *hTnf*, (i) *hIl1a*, (j) *hIl1b* and (k) *hil1rn* are shown. Data are pooled from 3 independent experiments. Data are shown as mean with SEM. (a-c, e-j) one way ANOVA. (d) Kruskal-Wallis test with Dunn's multiple comparison **$p \leq 0.01$, ***$p \leq 0.001$, **** $p \leq 0.0001$.

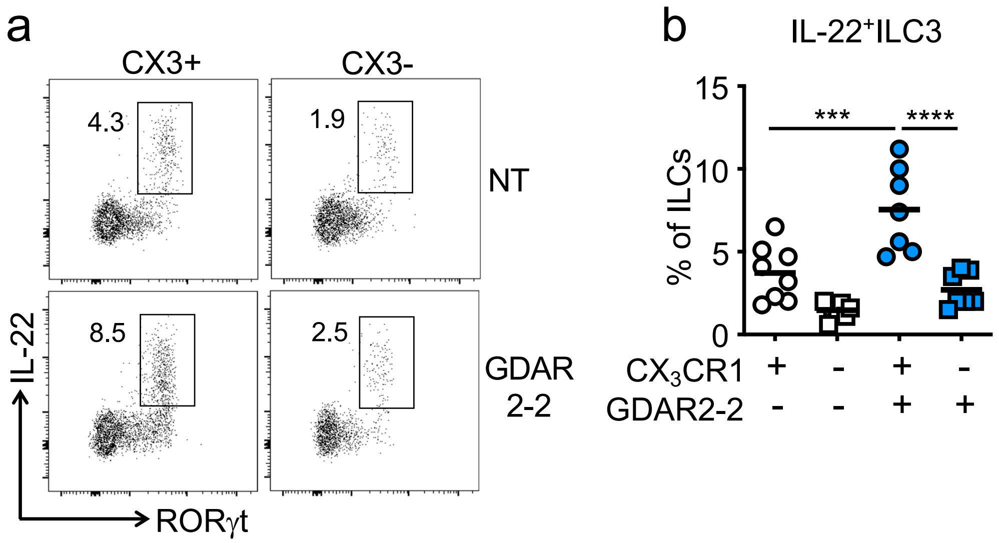

Supplementary Figure 7. CX_3CR1^+ MNPs are essential for GDAR2-2-induced IL-22$^+$ILC3 expansion. ABX and DT-treated littermate control (CX_3CR1+ , CX_3CR1^+ MNP sufficient) and CX_3CR1-DTR (CX_3CR1-, CX_3CR1^+ MNP deficient) mice were colonized with or without GDAR2-2 and infected with *C. rodentium*. 5 days after infection, cells from large intestine laminar propria were isolated and analyzed by flow cytometry. (a) Flow plot of live lin$^-$Eomes$^-$CD90$^+$ cells. (b) Percentage of IL-22$^+$ILC3s. Data are representative of at least 2 independent experiments. (b) Data points are single mouse with mean. One-way ANOVA with Bonferroni correction. *** $p \leq 0.001$. ****$p \leq 0.0001$

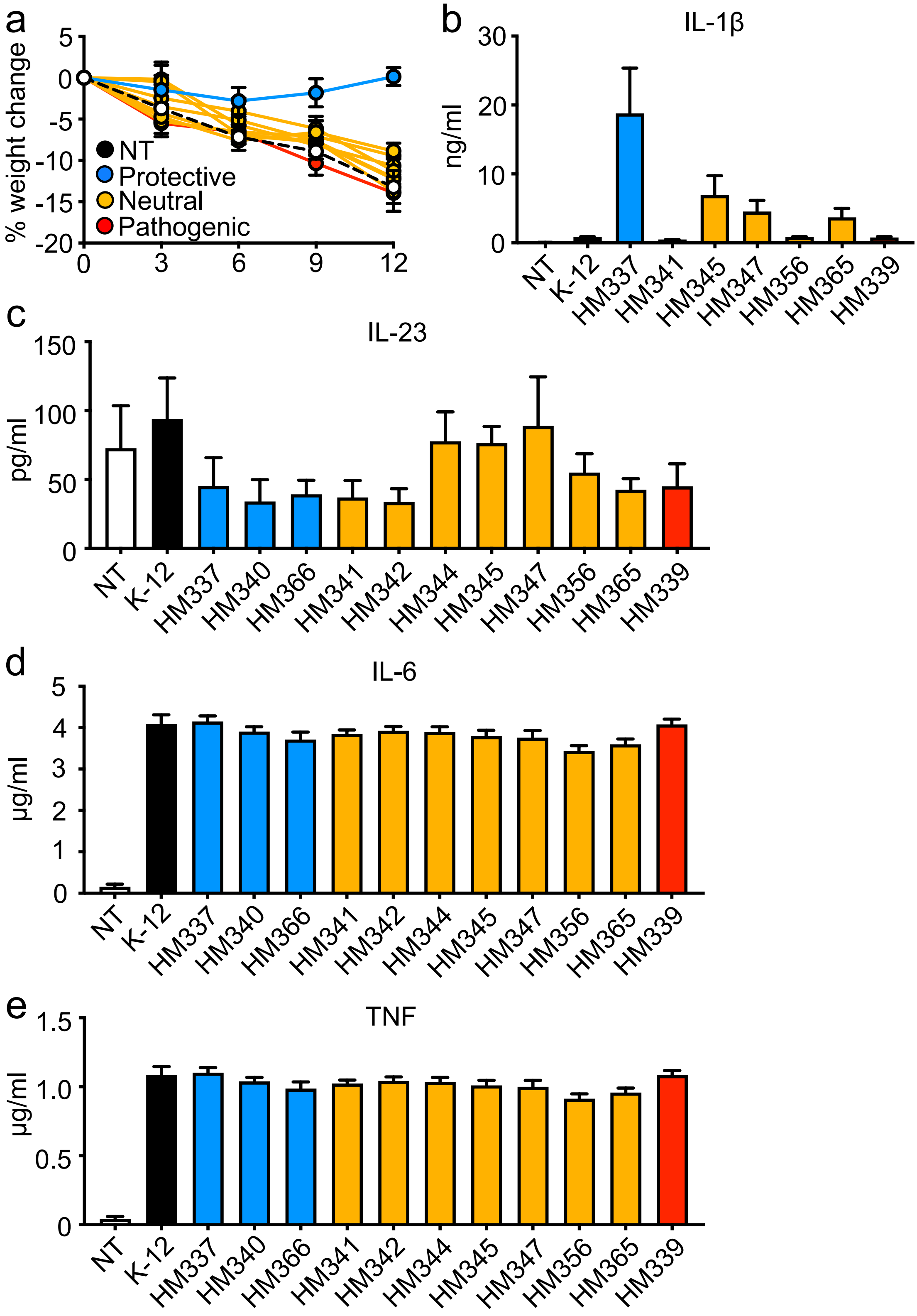

Supplementary Figure 8. Effect of protective and neutral *E. coli* for protection from *C. rodentium* infection. (a) ABX-treated B6 mice were colonized with indicated human *E. coli* isolates or left untreated and infected with *C. rodentium*. Weight changes are shown. (b-e) iBMDM were co-cultured with indicated *E. coli* isolates or left untreated. Supernatant analyzed by LegendPlex for cytokine secretion as indicated. Data are (a) representative of at least 2 independent experiments or (b-e) pooled from 3 independent experiments. No significant differences between groups was observed.
